# Adult newcomers’ perceptions of access to care and differences in health systems after relocation from Syria

**DOI:** 10.1186/s13031-022-00457-x

**Published:** 2022-05-21

**Authors:** Nancy Carter, Sandra Carroll, Rawan Aljbour, Kalpana Nair, Olive Wahoush

**Affiliations:** 1 School of Nursing, McMaster University, 1280 Main Street West – HSC 2J27, Hamilton, ON L8S 4K1 Canada; 2 Oakmed Family Health Team, Oakville, ON Canada

**Keywords:** Health services accessibility, Chronic disease, Refugees, Syria, Focus groups

## Abstract

**Background:**

In Canada, approximately 13% of the population lives with multiple chronic conditions. Newcomers, including refugees, have the same or higher risk of developing chronic diseases as their host population. In 2015–2016, Canada welcomed almost 40, 000 newcomers from Syria. This study aimed to (1) understand adult newcomer health needs for self-management of non-infectious chronic conditions; and (2) identify strategies to improve access to health care services to meet these needs.

**Methods:**

This study used a qualitative descriptive design. Interviews and focus groups were conducted with consenting newcomers, service providers and community agency administrators. Interview guides were developed with input from community partners and snowball sampling was used.

**Results:**

Participants included 22 Syrian newcomers and 8 service providers/administrators. Findings revealed the initial year of arrival as one of multiple adjustments, often rendering chronic disease management to a lower priority. Self-care and self-management were not routinely incorporated into newcomer lives though community health agencies were proactive in creating opportunities to learn self-management practices. Gaps in access to care were prevalent, including mental health services which typically were not well developed for trauma and post-traumatic stress disorder (PTSD), particularly for men. Newcomers expressed frustration with lengthy wait times and not being able to access specialists directly. Youth frequently played a key role in translation and disseminating information about services to their families.

**Conclusion:**

Chronic disease management was a low priority for newcomers who were focussed on resettlement issues such as learning English or finding work. Provision of practical supports such as bus tickets, translation, and information about the healthcare system were identified as means of improving access to care.

## Background

In Canada, approximately 13% of the population lives with multiple chronic diseases/conditions [1]. There is evidence that the prevalence of chronic disease is increasing in developing countries [2], with newcomers at the same or higher risk of developing chronic diseases compared to their host population [3]. Syria’s health system was similar to other middle-income countries prior to conflict starting in 2011 [4]. A combination of public and private health service delivery was in place, however, this led to inequities between urban and rural areas with the latter being underserviced [4]. By 2010, life expectancy had risen to 75.9 years. Despite improved mortality for both adults and children, cardiovascular disease, COPD, diabetes, and hypertension were highly prevalent in Syria [4]. A survey of newcomers from Syria to Jordan found a biologically based prevalence of diabetes and hypertension to be 19.3% and 39.5% respectively, for adults 30 years or older [5]. In this study, about one third reported missing medications in the past month, highlighting barriers to medical management [5]. A 2022 systematic review of pooled prevalence of non-communicable disease of refugees from Syria in neighboring countries found primary care rates of diabetes and hypertension to be 48% and 35%, and community rates to be 12 and 24% respectively [6]. These studies affirm a notable chronic disease burden in Syrians who have been displaced.

Access to chronic disease management and support is challenging for newcomers for a variety of reasons. In general, existing policies and services tend to place attention on mental health supports and communicable diseases [7, 8]. As well, connecting with appropriate health care providers and specialists can be challenging because of cultural and language barriers, difficulty processing access to insurance [9, 10], lack of healthcare provider capacity [11, 12], and complex health care needs [9, 11]. As a result, some newcomers tend to utilize emergency departments and walk-in clinics as their primary contact to access a healthcare provider [9, 13]. This disjointed care results in a reduced ability to provide prevention education and appropriate management of existing chronic disease [13]. An additional concern is the often prolonged interruption of self-management for chronic health conditions and fractured access to health care and supports during the refugee journey from country of origin to permanent resettlement [14, 15]. For persons who received care in their country of origin, this interruption could lead to the advancement of existing chronic conditions and negative health outcomes [16].

In 2016, Canada responded to a global call to accept refugees (herein named as newcomers) from Syria, Iraq and Afghanistan by committing to accept 25,000 newcomers in one year. By the end of 2016, this number reached 39,000. Community support for newcomers in many areas was unprecedented with multiple agencies and private sponsors working on resettlement. The Resettlement Assistance Program (RAP) funded by the Federal government, provided one year income support for those meeting the definition of Government-Assisted Refugees (GARs), which were most newcomers from Syria. This program includes case management, housing assistance, support to get children into school and adults to language training, and health navigation. After this year, newcomers moved to provincially funded social support programs. All newcomers from Syria were eligible for the Interim Federal Health Plan (IFHP) for up to one year, which included supplemental and prescription drugs benefits, as well as basic health coverage from the Ontario Health Insurance Plan (OHIP). However, changes to this program [17] may have resulted in confusion and hesitancy from health care providers, uncertain about what services were eligible under the IFHP.

Data from Citizenship & Immigration Canada, Immigrant Medical Exam (IME) report that the top chronic health conditions for Syrian newcomers include hypertension, diabetes and cardiovascular disease [18], all of which require ongoing management for quality of life. This aligns with predominant health concerns prior to the start of conflict [4]. This study sought to conduct a multi-stakeholder needs assessment involving newcomers, health care providers, and community stakeholders to systematically identify and understand factors that influence the ability of adult newcomers with non-infectious chronic diseases to access specialized services and manage their health.

## Methods

### Design

This study employed a qualitative descriptive design [19]. In keeping with the core principles of a qualitative descriptive design, sampling was purposive [19], and data collection centered on informational power which focusses on the quality of information within a sample.

The primary study objectives were to: (1) understand adult newcomer health needs for self-management of non-infectious chronic conditions, and (2) identify and compare patient, health care provider, and community stakeholder perceptions of unmet health needs related to management of non-infectious chronic diseases (e.g., diabetes, hypertension, chronic obstructive pulmonary disorder (COPD)), and strategies to improve access and delivery of health care services to meet these needs.

### Theoretical framework

A framework developed to understand immigrant health service utilization provided a theoretical underpinning to our methods [20]. This framework builds on previous health service utilization models and identifies special factors influencing the immigrant experience.

### Recruitment

Recruitment methods were multifaceted and included the following: advertisements in English and Arabic placed at locations accessed by newcomers and use of snowball sampling to connect providers and administrators. Advertisements for newcomers asked for participants who had health conditions such as heart diseases, diabetes, arthritis, or breathing problems and were interested in speaking about experiences getting healthcare in Canada. Participants signed a study consent form, which was reviewed in Arabic in all newcomer focus groups.

For both individual interviews and focus groups, the main inclusion criteria included being over the age of 18 and able to speak either English or Arabic, as the research team included a team member who spoke Arabic (RA). Newcomers were to have a chronic condition which was indicated on the recruitment poster and consent form, though not validated beyond newcomer self-report.

We conducted interviews and focus groups with: (1) Syrian newcomers who self-identified as having a chronic conditions; and (2) health and service providers and administrators from community health agencies working with newcomers. Ethics approval was obtained from the Hamilton Integrated Research Ethics Board (HiREB #3344).

### Data collection & analysis

Interviews and focus groups were held in community locations that were accessible to participants and were conducted by PhD trained researchers with expertise in qualitative research methods (NC, SC, KN), and RA, a research team member who spoke Arabic. Where necessary, a translator was used, and notes were taken during the group along with a digital recording so that it could be transcribed. The interview guides were developed with input from community partners. Questions for newcomers focussed on healthcare needs, access, and chronic disease self-management. Questions for community staff and health care providers focussed on service provision, barriers to access, and types of health issues encountered. All transcripts were de-identified.

Data analysis focussed on inductively deriving themes [19], that aligned with the Immigrant Health Service Utilization Framework [20], which was developed to address the unique health utilization context of immigrants. Two research team members were involved with the initial coding and analysis (NC and KN) to develop the codebook. Both team members reviewed the transcripts using the developed coding scheme, and one team member drafted the preliminary synthesis [19] (KN) which was then reviewed by the other team members (NC, SC, OW). Data was synthesized based on the four main elements of the Immigrant Health Service Utilization Framework: need for health care, resources, predisposing factors, and contextual/macrostructural conditions. Need for health care describes the conditions that prompt health care utilization. Resources relate to factors that support or inhibit health care utilization and include financial, social, and health care access. Predisposing factors are individual demographic factors that impact health care utilization such as gender, age, ethnicity, socio-economic status, marital status, health beliefs and attitudes, and immigrant status. Finally, contextual/macrostructural factors are broad government and policy level factors that impact health care utilization. It is recognized that all 4 factors can directly or indirectly influence each other as mediating effects.

## Results

Overall, 30 participants took part in an interview or focus group, which included 22 newcomers and 8 health care providers and administrators. There were two female newcomer groups (n = 6; n = 11), and one male group (n = 5). Specific demographic information was not collected from newcomers in an effort to minimize paperwork in the group and focus on hearing participants’ experiences. Based on interviewer feedback, it was apparent that the majority of participants were in their 20’s–30’s with school age children.

As well, there was a group with two providers, four individual interviews with providers, and two individual interviews with community stakeholders. The providers included a range of professionals: nurse practitioner, occupational therapist, physical therapist, registered nurse, and community workers. The two community stakeholders held administrative positions in local community health agencies serving newcomers.

Supporting quotes provided below include the interview/focus group number, with newcomer groups distinguished by gender (male or female), and providers and community stakeholders denoted as providers. Data are synthesized based on the elements of the Immigrant Health Service Utilization Framework [20].

### Need for health care

Healthcare needs can be divided as general needs or immigrant specific needs [20]. Newcomers experienced a range of health conditions and illnesses, including both acute and chronic illnesses. Diabetes was discussed and sometimes newcomers had gaps in understanding the symptoms of their diabetes. For example, one support worker noted how a newcomer with diagnosed diabetes was unaware that the symptoms they mentioned could be related to their diabetes. Other chronic conditions included high cholesterol, high blood pressure, anxiety, thyroiditis, neuropathy, heart issues, and aneurysm. Some acute conditions included dental issues, burns, cataract, knee problems. Pregnancy care and children’s health concerns were also noted. Newcomers mentioned the types of health conditions experienced by themselves or family members but mainly discussed the challenges with accessing healthcare which related to long wait times and inability to access specialists directly. Although non-infectious, chronic conditions were mentioned, this was not a predominant concern or priority for newcomers. Further, the concept of self-management was not familiar for most newcomers. One newcomer noted that he “*self medicates for ongoing psychological health challenges”* (FG3).

### Resources

Overwhelmingly newcomer participants experienced challenges and difficulties in accessing the health care that they wanted for *both* acute and chronic health care conditions. Specific to immigrant utilization, homeland-based financial and social resources, and transnational access to healthcare influence access to health care.

Lack of affordable transportation and limited finances impacted the ability to care for chronic illnesses. Newcomers found that fruits and vegetables were more expensive in Canada. Although some newcomers paid for services such as a private nurse or translation services, they reported that they felt these services would have been covered.

Language barriers created frustration for newcomers as they were often left feeling that their provider was not invested in their care which created a scenario where some only accessed a provider if their medical concern was serious. Some newcomers experienced an element of discrimination in their engagement with the health care system in Ontario, particularly with specialists. One participant inferred that this indicated discrimination: “Once they know you are Arabic speaking, they refuse requests.”(Female 1). Further, there was sometimes a discomfort in disclosing information when a translator was present for fear information would be repeated in their community.

Language barriers were overwhelmingly cited by providers as the main barrier for newcomers to access health care. The lack of understanding and availability of translation support by providers and specialists was a deterrent to accessing care. Poor language skills also created issues for primary care for all family members. For instance, a provider reported newcomers were unable to read letters from Public Health indicating that their children needed vaccinations. Although newcomers were not opposed to getting their children vaccinated, they could not comprehend the notices. Clients often brought mail to appointments and asked for assistance in understanding what was being sent to them.

Language difficulties also led to confusion after appointments with health care providers, and often other support workers or providers helped with translation. One support worker described this issue and what they do to help, despite not having a medical background:

A lot of these clients are walking away not really understanding what they have, and that’s huge. And then coming to outside help, people like me or social workers or whoever works with the family, and, hey what’s going on with you? Let’s Google it together. Because I’m not in the medical profession, you know what I mean?(Provider2)

If a translator was not available, newcomers typically relied on translation apps on their phone. One participant described potential problems with this: *“If they’re unable to get a translator to the appointment they’re relying on Google Translate, if they have data on their phone. And then there’s a lot of things that get lost in the mix. They’re unable to express themselves in the ways that obviously they’d be able to do in their first language. (Provider2).*

Lack of financial resources to support transportation was also noted as a challenge. The provision of bus tickets or taxi fare to and from visits was an incentive for newcomers to attend appointments. This practice was not consistent however and differences at various health and social service agencies, led to confusion and uncertainty for newcomers. Here, some providers were aware of resource constraints and made efforts to ameliorate this impact.

### Predisposing factors

For immigrants, predisposing factors that can influence health service utilization include immigration status, assimilation, and immigrant ethnic culture [20]. Providers were particularly attuned to various predisposing factors (e.g., gender, health attitudes) which could impact health care utilization. Providers reported that the initial year of arrival for newcomers was one of adjustment with many competing priorities, often making chronic disease management a lower priority. Newcomers experienced a range of issues that impacted their ability to maintain their health. This sometimes resulted in a disconnect between patient needs and provider goals. A Nurse Practitioner said:

Our priority is managing your chronic disease, getting control of those things, but that’s not someone else’s priority and that is a difference in communication. I think we’re trying to communicate what our concerns are and, that’s not their concern right now. (Provider6)

Interestingly, the providers that were interviewed also highlighted some gaps in understanding the newcomer experience by specialists who did not have connections with the primary care system.

Providers reported broader concerns such as family reunification which caused considerable stress for newcomers as they worried about their family being separated. Many newcomers also came to Canada having experienced trauma prior to arrival and often did not have the language and understanding for how to express and manage their trauma. In addition, mental health and the number of chronic conditions experienced were barriers to maintaining health. A physiotherapist described the impact of mental health issues on self-management:

I’d say mental wellness can be a barrier. It’s quite a big barrier if someone is feeling very depressed, isolated, and having very low mood, low energy levels, that they won’t access services. And then I would say in general, the number of chronic conditions someone has, the more challenging it is to come to appointments. Because they may miss something because of their pain or because of something else or they may just have way too many appointments to go to, so that can be a barrier to accessing. (Provider3)

This provider also noted there should be a focus on the strength and resiliency of newcomers and said this: *“I think … to highlight their strengths and their resiliency and how they’ve made it. These are really tough people. They’ve made it through really hard life situations. And they’re doing the best that they can…’ (Provider3).*

It was reported that there were gaps in services related to mental health, and where available, these providers were not appropriately trained to address trauma and PTSD, particularly for men. One provider who provided programming for men commented on this:

A lot of my male clients will talk.… I do find that the services out there lack that understanding of the situations that they may have come from. So, trauma, but they (providers) don’t understand the trauma that’s happened, and they can’t comprehend it. And that is the feedback from my clients. (Provider7)

The health of children and teenagers sometimes was not a prime concern as parents and health care providers struggled to manage their own issues and concerns. One provider noted, “*You know it’s easy for children to fall on the back burner if there’s somebody else with more pressing health issues or something else that going on. And mom and dad can only do so much.” (Provider2).*

Providers suggested it might be a cultural issue that some families did not realize the importance of self-care, taking medications, or taking time off from work to care for themselves. They noted that some newcomers had a different perception of the responsibilities in chronic disease management, with self-management sometimes an unfamiliar concept. She noted, *I’m not here for you to sit here, and for me to fix you. You have what it takes to heal yourself, and I’m here to support you and guide you through that. (Provider3).*

Women were less attuned to their own health needs, though centres created opportunities for women to come together and become informed about their health, including self-management. Strategies included women-only exercise classes, which allowed women take part in a self-management activity, while also learning about local services and supports. A physiotherapist described the benefits of the exercise class for newcomer women who have complex needs:

…just women starting to enjoy it and recognize that it’s good for their bodies and starting to do the elements at home. But also, self-care, because for a lot of these women, a big piece of their pain experience is just a lack of time to care for themselves. Being more of a group- focused culture than an individual-focused culture, there is the sense women care more about their family than themselves and that’s common. And for a lot of these women, a lot of their family are still in refugee camps. And so, there’s this ongoing sense of tension and fear for their children so it makes sense to me that they’re not well because they’re so worried about their kids and they don’t know when they’re going to come. (Provider3)

Programs like this, though, were dependent on having appropriate translation services available. Providers reported youth who learned English in school played a key role in disseminating information about services to their families and often took on the role of interpreter or translator.

Stakeholder participants were administrators who worked in community agencies that organized health and social support for newcomers. They described the stress and impact of previous hardships newcomers endured related to conflict in their home country and migration. Language difficulties were also raised by stakeholder participants. Stakeholders were mindful of contextual factors and often took on advocacy roles to inform other groups about the needs of newcomers.

Stakeholders noted that a range of services were available in the region to support newcomer health in a holistic and comprehensive manner, both for chronic and non-chronic conditions.

### Macrostructural/contextual factors

The context of emigration, context of reception and health service utilization in the homeland are all macrostructural/contextual factors that influence health service access by immigrants [20]. Contextual factors related predominantly to newcomers’ experience in a health care system very different from that in Canada. Many were frustrated that they needed a referral from a family physician to see a specialist and with the long wait times once a referral was made. Newcomers were accustomed to accessing specialists on their own and often seeing them on the same day. One could also go straight to a pharmacist to obtain some medications compared to Canada where you need to see a physician for a prescription. As such, they experienced significant adjustments to the referral and appointment system in Ontario, Canada. In Canada, however, newcomers did not have to pay for vision testing and glasses which they would have in Syria.

The methods of interaction and communication with health care providers were also different from what newcomers were accustomed to. As noted by one newcomer, the “*process of communication is different, where they are used to hearing things can get better, vs 'this is getting worse’”. (Female1)*. Another newcomer commented on the difference in communication from providers in Canada and said, *“In Syria you would not expect to be told directly that you have cancer; you would tell someone close to them and let them calm down and then let them know.”(Female1).*

Community stakeholders acknowledged the challenges of keeping newcomer issues and concerns at the forefront, with systemic issues of how refugee claimants are funded, how agencies are funded, and shifting local health priorities impacting this. Said one administrator:

I would hope that there aren’t as many barriers that individuals are facing, that we do get better at lessons learned so that the good things that come out of it have some sort of sustainability. And I think that does come with communication and accountability. But if we all get siloed off, we all get diverted to, this is our new thing and in an ever-changing healthcare system, that happens. (Provider1)

Changes to IFHP further influenced the types of supports that providers and agencies were able to recommend and prescribe.

Stakeholders also recommended a more standardized approach to health care delivery for newcomers, emphasizing the multiple different agencies involved in newcomer support which often caused disconnects for providers and patients. While there were many examples of agencies working together to meet the needs of newcomers, the reality of limited funding and mandates created a scenario where some agencies were seen as the main providers of care for newcomers, resulting in others not taking an active role in adapting to the needs of newcomers. Participants stated that continued collaboration was needed. For example, one community stakeholder said “*I think it’s great we are able to come together when needed. It would be great if it was something that was ongoing and there was an accountability and people all stepped up and said what can we do, so that others aren’t feeling the burden." (Provider1)* Agencies that worked with newcomers were often smaller with less funding compared to other organizations, further contributing to inequitable burdens of care.

The importance of educating the next generation of health care providers on issues related to newcomers was also raised, with the suggestion of purposeful exposure of trainees to newcomers and their healthcare needs: “… *if we’re wanting to encourage the next generation of providers to pick up the work and to close the gap, it’s important that they have access to it in a very strategic and structured way [that exposure] so learners are able to lead clinics. (Provider5).*

Some newcomer participants felt that they were not getting appropriate support or referrals, and gaps remained regarding what services were available to them. For example, one patient noted that they had been “*asking to see an endocrinologist for 3 years and the family doctor won’t refer*” (FG2) Long wait times created uncertainty, with worries that their health was worsening as they waited.

Despite these frustrations, most newcomers were generally pleased with the care they received once they accessed healthcare. Newcomers appreciated that there were some brochures available in Arabic, and noted wanting more information by phone (e.g., Arabic messaging). Newcomers also stated that they received health information from friends, through classes taken at the clinic, their family doctor or specialists, and health news from television.

Taken together, the voices of newcomers, providers, and stakeholders, offer a clear direction for how to better support newcomers with chronic disease for their self-management.

## Discussion

This descriptive qualitative study sought to understand Canadian Syrian newcomer heath needs for self-management of non-infectious chronic conditions. We found that chronic disease self-management was a low or non-existent priority for the newcomers that were interviewed because of competing priorities related to resettlement. While self-management and self-care were typically not part of the health lexicon for newcomers, it is noteworthy that community agencies were creative and proactive in attempting for address chronic disease self-management. These efforts suggest that newcomers are open to learning about self-management and while it can be challenging to address this early in the resettlement process, newcomers are interested in taking care of their chronic diseases. Further, ease of access to medications and specialists in their home country may have assisted with previous self-management practices. The Immigrant Health Service Utilization Framework was useful for understanding the newcomer experience of healthcare and highlighted the essential role of support agencies who are attuned to predisposing factors (e.g., gender, cultural norms) which influence the types of care newcomers’ access.

Concerns about mental health were a common theme in our findings. The presence of mental health concerns and disorders has been documented in other research with refugees from Syria [21, 22]. A recent systematic review examining mental health and mental health supports for refugees from Syria living in neighbouring countries found that post-traumatic stress disorders ranged from 16 to 84%, with anxiety and depression also being commonplace [21]. As well, though there has been research examining the experiences of mental health issues of women from Syria [22], the experiences of men have not been specifically elucidated. Our study has highlighted the particular concerns of men, suggesting that more focussed effort in this area may be needed. Gaps in care could be addressed through the use of a transcultural lens in trauma informed care [23].

The second aim of this study was to identify strategies to improve access and delivery of healthcare services. Access to timely healthcare was a concern universally noted by newcomers, which has been previously documented with newcomers stressed by wait times to see specialists and lack of access to services such as dental and pharma care [24, 25]. Language barriers created challenges with access to health care and chronic disease self-management. Some newcomers relied on translation apps on their phone to manage appointments when translation services were not provided by the service provider that they were seeing.

Navigation within the healthcare system was essential for newcomers who benefited from providers who purposefully shared strategies such as asking to be put on a cancellation list when wait times are long. The community providers interviewed were aware of their role in imparting this information and seemed to take care in ensuring that these strategies were shared. One of the main challenges for many newcomers, however, was the limited time they received newcomer focussed services, and the transition towards non-newcomer supports. Continued assistance with health system navigation could help with ameliorating these barriers to healthcare access.

One new area that arose from these interviews was the discussion around health professional education. Agencies that incorporated health care trainees who worked with newcomers felt this that was critical healthcare education for trainees. This exposure to the social determinants of health specific to newcomers was seen as a step towards having an aware and sensitized future generation of health professionals.

We found that there were a number of key drivers that could support the movement of adult newcomers from a focus on survival to the acknowledgement and attention on self-management for chronic disease. First, community health agencies were attuned to what could facilitate this shift and proactively had these supports in place (e.g., women-focused exercise classes). Second, explicit education about how the Canadian healthcare system is organized and how to improve access helped to support newcomers’ efforts at self-management. Finally, there was a respect for newcomers’ priorities and, as such, an understanding that there were other priorities that may necessitate attention prior to chronic disease self-management.

### Strengths and limitations

This study has yielded a number of actionable strategies that could assist newcomers with chronic disease self-management. The inclusion of varied perspectives was helpful for triangulation of findings. As well, the use of the Immigrant Health Service Utilization Framework allowed for an immigrant-focussed lens for the data collection and analysis. A number of limitations are worth noting. First, we conducted this study in one, urban city where healthcare coordination may be different from other cities and rural settings. As well, our participants were recruited from community-based agencies, resulting in the omission of hospital and specialist perspectives, two key areas where chronic disease support takes place. Despite the utility of the Immigrant Health Service Utilization Framework, its generic focus on the immigrant experience did not capture the unique challenges faced by refugees. For example, specifically highlighting the need for mental healthcare and exploration of trauma associated with violence and upheaval would be important. Future research could include a focused adaption of this framework for refugees. As well, the inclusion of a broader representation of providers (e.g., specialists) would add to this data, particularly as challenges interfacing with these groups were noted.

## Conclusions

Chronic disease management was often a lower priority for newcomers who were more focussed on resettlement issues such as learning English or finding employment. Provision of practical supports such as bus tickets, translation services, and information about how the Ontario healthcare system works were identified as means of improving access to care. From a community perspective, administrators and health service providers noted the importance of early integration of health professional learners into healthcare services to build capacity as well as ongoing engagement and accountability from all health care agencies within a region. With Canada focused on targeting greater numbers of refugees, these strategies can help address some of the current gaps experienced by newcomers.

## Data Availability

The data generated from this study are not publicly available in order to protect participant privacy.

## Abbreviations

PTSD: Post-traumatic stress disorder
RAP: Resettlement Assistance Program
GAR: Government-Assisted Refugees
IFHP: Interim Federal Health Plan
OHIP: Ontario Health Insurance Plan
IME: Immigrant Medical Exam
COPD: Chronic obstructive pulmonary disease
HiREB: Hamilton Integrated Research Ethics Board
